# Open questions on the chemical composition of airborne particles

**DOI:** 10.1038/s42004-020-00347-4

**Published:** 2020-08-07

**Authors:** Barbara J. Finlayson-Pitts, Lisa M. Wingen, Véronique Perraud, Michael J. Ezell

**Affiliations:** Department of Chemistry, University of California, Irvine, CA 92697-2025 USA

**Keywords:** Environmental monitoring, Atmospheric chemistry, Mass spectrometry

## Abstract

Airborne particles have significant impacts on health, visibility, and climate. Here, an overview of what is known about particle chemical composition is presented, along with open questions and challenges that are central to relating composition to life cycles and impacts.

Airborne particles have been known for at least 800 years to negatively impact visibility and health. For example, Moses Ben Maimonides (1135–1204) described air in Cairo as “stagnant, turbid, thick, misty, and foggy”, and attributed “dullness of understanding, failure of intelligence, and defect of memory” to “spoilage of the air”. A recent estimate is that exposure to outdoor particulate matter (PM) currently leads to 3.3 million premature deaths per year worldwide, and this could double by the year 2050^1^. It is known that these particles impact climate as well^2^. The chemical composition of the particles is an important determinant of all of these atmospheric impacts. Hence, understanding the composition and processes affecting composition is critical for developing cost-effective control strategies.

A first step is to distinguish “primary particles”, those that are directly emitted from natural and anthropogenic sources such as sea spray, transportation, industry, forest fires, etc. from “secondary particles”, those formed from chemical reactions of gaseous precursors in air. The latter include both inorganics such as sulfuric acid/sulfate from SO_2_ oxidation and nitrate from NO_x_ oxidation, as well as a host of volatile organics, which make up a major fraction of the particles around the world^3^. Because there are thousands of potential organic precursors in air, and a number of different oxidation reactions involving O_3_ and OH, Cl, and NO_3_ radicals (Fig. 1), the organic component of particles becomes very complex^4^. Further adding to the complexity of this secondary organic aerosol (SOA) are reactions in the condensed phase that can also form new products after the particle has formed^5^.

**Fig. 1:**
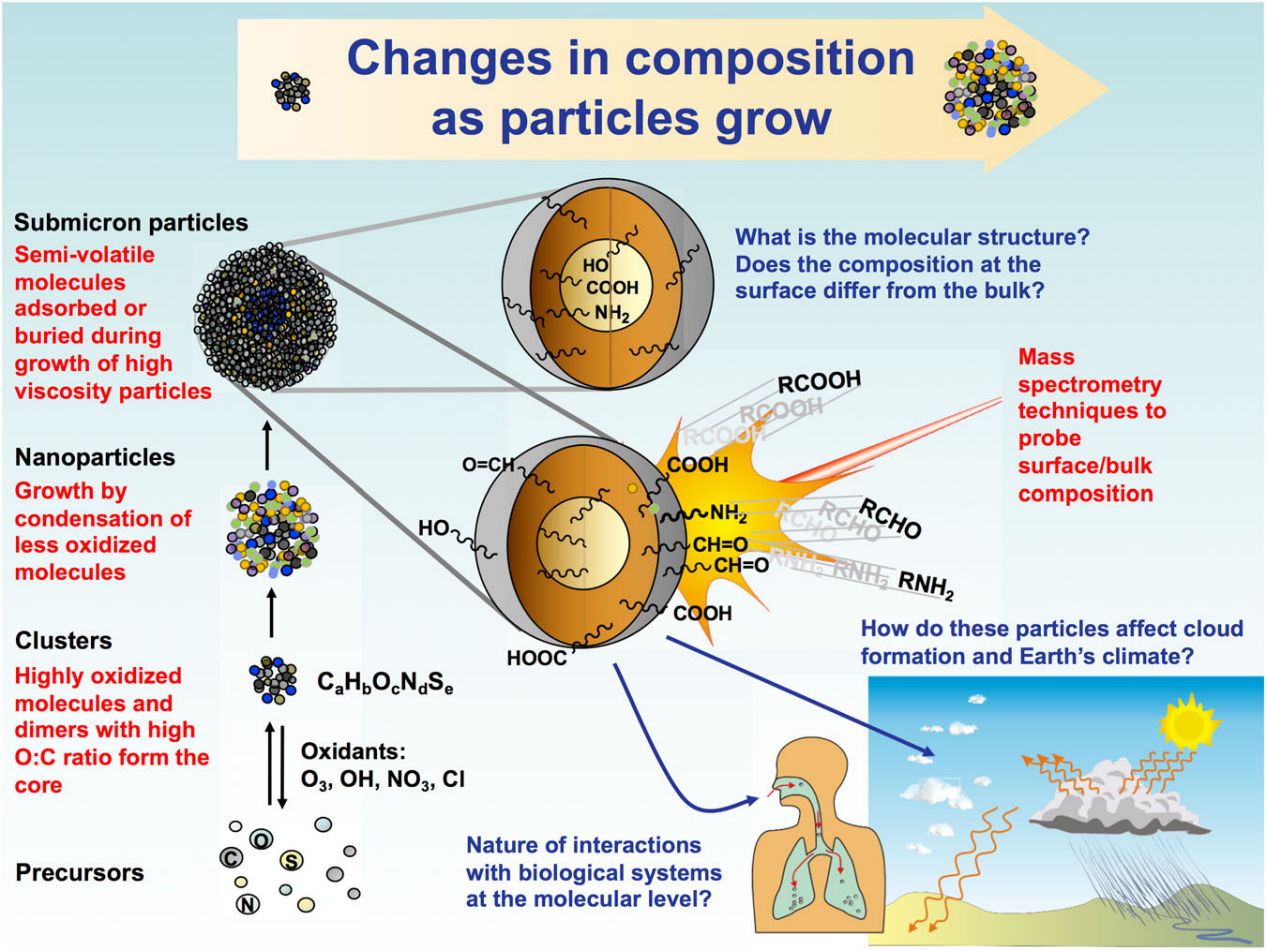
Schematic of the life cycle of secondary organic aerosol particles and their impacts. Volatile organic precursors are oxidized in air by O_3_, OH, NO_3_, or Cl, and the resulting highly oxygenated, low-volatility products formed from these reactions nucleate new particles, and/or condense on pre-existing ones to grow them. Different species participate in particle growth at different stages of the life cycle, with less oxygenated, more volatile species condensing on the larger particles. Chemical composition of these SOA particles will depend on the environmental conditions surrounding the particles (relative humidity, temperature, gas phase composition …etc.), as well as their physical properties (i.e., viscosity) governing the uptake of trace gases. Once they reach ~50–100 nm, these particles are able to impact climate directly by interacting with incoming solar radiation but also indirectly via cloud processing. The chemical composition and size of these particles will also determine their impact on health.

## Composition, size, and phase

A critical property of atmospheric particles is their size. Particles must grow from sub-nm seeds formed by the gas phase chemistry to ~100 nm in order to scatter light efficiently. This reduces visibility and affects climate both directly through light scattering and indirectly through their impacts on clouds. This ~100 nm size also efficiently reaches the alveolar region of the lung. Most importantly, the chemical composition of particles changes during the life cycle of particle formation and growth, reflecting changes in the processes that occur at each stage^6^. For example, the O:C ratio of particles increases as reactions with atmospheric oxidants such as OH and O_3_ occur^4^. The composition also changes due to photolysis, hydrolysis and condensed-phase reactions that can, for example, form new light-absorbing species such as brown carbon^7,8^. Measuring particle composition from the nm to micron scale is thus important for understanding particle nucleation, growth, physicochemical transformations and impacts (Fig. 1).

It had been assumed for many years that secondary organic particles were low-viscosity liquids, so that exchange with the gas phase and diffusion inside the particles (which facilitates gas uptake and in-particle chemistry) was relatively rapid. However, over the past decade, it has been demonstrated that under many conditions, particles are high viscosity solids or semi-solids^9^. In that case, uptake from the gas phase by diffusion into the particle following adsorption at the surface is slow, changing the mechanism of gas uptake that grows the particle and decreasing opportunity for in-particle chemistry^10^. In addition, the relative importance of uptake of gases into the particles may be different for high viscosity versus liquid particles, impacting the particle chemical composition. Both the phase/viscosity of particles and the chemical composition are very sensitive to the environmental conditions, including temperature, relative humidity, and the gas phase composition during particle formation, particularly the concentrations of NO_x_.

## Overview of chemical composition of particles

Given the plethora of different compounds in particles, and particularly the organic component, it is perhaps not surprising that a complete molecular identification of all components has not been achieved. There is no one technique that provides a comprehensive analysis of all inorganic and organic species, and most approaches have been targeted to subsets of the components in limited size ranges. Elemental analysis shows C, H, O, N, S as well as trace metals are very common components, and it is known that sulfate, nitrate, and ammonium ions as well as organics are ubiquitous in particles^3^. The organic component of primary particles tends to be less oxidized (e.g., polycyclic aromatic hydrocarbons in soot) and can contain unique compounds that serve as tracers of specific sources. The organic component of SOA is even more complex, including many different oxygenated species (e.g., acids, alcohols, aldehydes and ketones, esters, peroxides, aldols, hemiacetals, and acetals) and nitrogen- and sulfur-containing organics (e.g., alkyl- or heterocyclic amines such as imidazoles, organonitrates, organosulfate esters). A growing number of measurements of SOA show that highly oxidized and high molecular weight oligomeric compounds with larger O:C ratios are relatively more important in smaller particles, whereas growth to larger diameters involves less oxidized, lower molecular weight species^4^.

## Approaches to determine the chemical composition of particles

Historically, and continuing to this day, particles have been collected using filters or impactors and the bulk samples collected in this manner have typically been extracted into a solvent and analyzed using chromatographic, spectroscopic, and mass spectrometric methods^11,12^. While very useful, the results are dependent on a number of factors such as the nature of the extraction solvent. Those components that are most soluble in the chosen solvent will be over-represented in the analysis and for some solvents such as methanol, there is a possibility of reactions (e.g., esterification of acids) occurring during extraction. Filtration methods tend to be biased toward larger particles that dominate the sample by mass. There is also the possibility of reactions of the particles on the filter during collection, particularly if oxidants are not removed from the air prior to filtration.

This led to the development of a number of on-the-fly mass spectrometric approaches for analysis of bulk composition of airborne particles or single particle analysis without the need for prior collection^7,8,12^. Mass spectrometry (MS) is a very powerful technique for providing both elemental information and in the case of high resolution MS, molecular formulae of complex, higher molecular mass organics. All of the on-the-fly approaches involve a number of steps: (1) sampling the particles in such a way that there is sufficient mass for detection; (2) transferring the particle components from the condensed into the gas phase; (3) ionizing the particle components; and (4) characterizing the ions using MS. The first step, concentrating the particles from air into a beam, is commonly accomplished using aerodynamic lenses. The second step typically involves either laser ablation or heating. In the case of laser ablation, lasers in the UV have most commonly been used, which efficiently vaporize the particles, including any metallic components and simultaneously causes ionization of the vaporized components, providing elemental composition. Alternatively, infrared lasers can be used for vaporization, followed by UV radiation for ionization. The high energies involved with UV laser ionization tend to cause extensive fragmentation of organics, with a few exceptions such as the polycyclic aromatic hydrocarbons. On the other hand, vaporization by heating is not effective for most metals but does preserve more of the molecular structures of organics with the exception of decomposition of thermally sensitive molecules.

In addition to laser ionization, there are a number of less-energetic approaches to ionization. Traditional electron impact ionization has the advantage that there is a wealth of data on the fragmentation patterns of many different potential particle components, and it can be less fragmenting/damaging than some UV ionization techniques. With the complex mixtures found in SOA there are so many different contributions to the mass spectrum that identifying parent compounds (those from the sampled particles that are ionized but have not fragmented) can be very challenging. In this regard, chemical ionization approaches have proven powerful for sensitive and molecular detection of specific classes of compounds^7,12^. Commonly used ionization reagents include protons in the positive ion mode and O_2_^−^, iodide, nitrate, and CF_3_O^−^ ions in the negative ion mode. Some more exotic reagents such as N-methyl-2-pyrrolidone have been recently used for measurement of species that can be difficult to detect such as ammonia in nanoparticles^13^. The use of elemental analysis with high-resolution techniques and factor analysis of mass spectra has notably increased our understanding of particle sources, level of oxidation, and the ubiquity of organic aerosol in air^3^.

A relatively new approach uses ambient ionization, which combines the transfer to the gas phase and ionization by intersecting a particle flow with a stream of charged droplets or a metastable plasma^12,14^. The former can be generated using different techniques such as electrospray or sonic spray. These techniques have the advantages of providing molecular information for organics, but sensitivity for the small mass of ambient levels of SOA is a challenge^15,16^.

## Key open questions and challenges

Despite the great strides we have made in elucidating key species responsible for particle formation and growth over the past several decades, there are many open questions and challenges remaining regarding the inorganic and the molecular organic composition of particles as a function of the nature of precursors and of the formation, growth, and aging processes that occur in air. These are driven by the need to address the impacts that particles have on health, visibility, and climate. For example, there is increasing evidence that ultrafine particles <100 nm in size may have disproportionate health effects^17^ that are not captured in their contribution to the total mass. The mass of such small particles is exceptionally low, making detection a major challenge, especially for measurements made on-the-fly. At present, air quality standards are currently set for the mass of particles <2.5 μm in diameter (PM_2.5_) and those up to 10 μm (PM_10_).

**Fig. 2:**
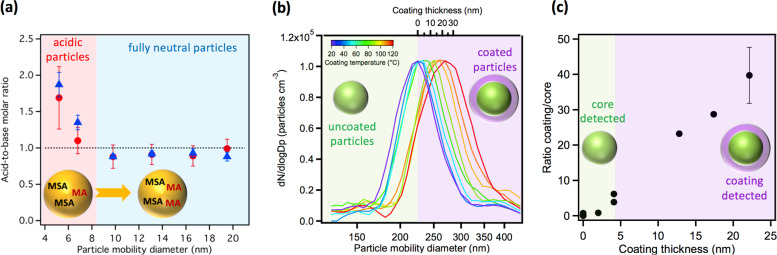
Probing the chemical composition of particles as a function of diameter. **a** Size-resolved bulk chemical composition of nanoparticles formed in a flow reactor from the acid:base initiated reaction between gas phase methanesulfonic acid (MSA; 2.3 × 10^11^ molecules cm^−3^) and methylamine (MA; 1.4 × 10^11^ molecules cm^−3^) under dry conditions. Particles were sampled at 0.6 s and 9 s residence time, respectively, using a thermal desorption chemical ionization mass spectrometer^13^; **b** size distributions of monodisperse 220 nm glutaric acid particles coated with malonic acid with coating thickness shown on top axis and **c** ratio of malonic acid (coating) signal to glutaric acid (core) signal detected for the particles in b using sonic spray ionization mass spectrometry^16^.

However, measuring the composition of these smaller (<100 nm) but more numerous particles remains largely an open issue^6,18^. Thus, although it is known that acid-base reactions play a significant role in new particle formation, this may not occur in a simple 1:1 acid:base stoichiometric fashion. For example, Fig. 2a shows the acid:base molar ratio for nanoparticles formed from the gas phase reaction of methanesulfonic acid with methylamine, where the smallest particles (<9 nm) contain more acid than base^13^. The capability of measuring chemical composition down to ~1 nm is crucial for understanding how stable clusters form and subsequently grow to detectable particles.

Another challenge is being able to measure all particle components, both inorganic (including metals) and organic simultaneously, with identification of specific organic molecules. The SOA portion in particular is known to contain many compounds that are fragile with respect to the vaporization and ionization processes used to detect them. Although elemental information such as O:C and H:C ratios can be obtained using a number of techniques, identifying specific compounds or even all of the functional groups remains difficult. For example, peroxides and hydroperoxides, as well as higher molecular mass compounds representing the reaction products of the backbones of two or more of the parent species (often referred to as dimers or oligomers) are commonly found in SOA^7,12^. Transferring such compounds into the gas phase without causing decomposition or secondary chemistry is a significant challenge.

A third challenge is analyzing particles not only as a function of size, but also as a function of their 3-D structure. If they are liquid and homogeneous, rapid diffusion results in well-mixed particles. However, this is not always likely to be the case. For example, liquid-liquid phase separation has been documented in both laboratory studies and field samples^19^ where aqueous and organic phases are immiscible. In addition, the mechanisms by which high viscosity solid and semi-solid particles grow will not be primarily by quasi-equilibrium uptake from the gas phase as is the case for liquid particles where solubility is a determining factor. In the high viscosity case, growth will depend on the interaction of the gas with the surface of the particle, and whether the attractive forces are strong enough to bind the gas to the surface for sufficient time to become incorporated into the particle^20^. The factors determining growth in this case are different from those for liquid particles, with the nature and composition of the particle surface driving uptake. This may lead to a composition that differs from that expected based on solubility considerations alone, but little is known regarding such potential differences.

MS techniques that can examine the molecular surface composition of such particles versus their bulk composition are on the rise and will help us to understand differences in composition between high viscosity and liquid particles. An example of one approach is shown in Fig. 2b, c, where laboratory-generated model glutaric acid particles were coated with increasing thicknesses of malonic acid and the ratio of coating to core signals measured using sonic spray ionization MS. As the coating thickness increases, less of the core is detected relative to the coating, with the probe depth estimated to be 2–4 nm^16^.

Just as major progress has been made over the last decades we can expect significant progress in the coming years. As has often been the case, the development of new analytical techniques that are sufficiently gentle and provide accurate molecular information will largely drive new understanding. In this vein, having techniques that can peel particles layer by layer “like an onion” could be quite valuable. It is sometimes the case that doing the measurement actually changes the sample during analysis, a sort of “atmospheric uncertainty principle.” Techniques that do not alter the sample composition during analysis will thus be important. Minimally destructive techniques will also help in tracing which products are formed by chemistry within the particle versus uptake from the gas phase. Finally, development and application of quantitative methods that cover the entire periodic table in an integrated approach over a wide size range is the ultimate goal that provides an enormous challenge for the coming decade. However, it is the essential basis of quantitative models that can provide a predictive capability for assessing particle impacts and developing effective control strategies.
